# Supplementary material for: HIVprotI: an integrated web based platform for prediction and design of HIV proteins inhibitors
Source: J Cheminform. 2018 Mar 9;10:12. doi: 10.1186/s13321-018-0266-y (PMC5845081; doi:10.1186/s13321-018-0266-y)
Supplement: Supplementary file 2 — Additional file 2. Source code of HIVProtI web server. [file 13321_2018_266_MOESM2_ESM.zip › HIVprotI_Source-code/hivprotI/weka-3-6-6/doc/weka/classifiers/IterativeClassifier.html]

IterativeClassifier


---


|  |  |  |
| --- | --- | --- |
| **Overview** \| **Package** \| **Class** \| **Tree** \| **Deprecated** \| **Index** \| **Help** \| **Weka's home** | |  |
| **PREV CLASS**   **NEXT CLASS** | **FRAMES**    **NO FRAMES**     **All Classes** |  |
| SUMMARY: NESTED \| FIELD \| CONSTR \| METHOD | DETAIL: FIELD \| CONSTR \| METHOD |  |


---


## weka.classifiers Interface IterativeClassifier

**All Known Implementing Classes:**: ADTree

---

``` public interface IterativeClassifier ```

Interface for classifiers that can induce models of growing
complexity one step at a time.

**Version:**
:   $Revision: 1.3 $

**Author:**
:   Gabi Schmidberger (gabi@cs.waikato.ac.nz), Bernhard Pfahringer (bernhard@cs.waikato.ac.nz)

---

| **Method Summary** | |
| --- | --- |
| `java.lang.Object` | `clone()`             Performs a deep copy of the classifier, and a reference copy of the training instances (or a deep copy if required). |
| `void` | `done()`             Signal end of iterating, useful for any house-keeping/cleanup |
| `void` | `initClassifier(Instances instances)`             Inits an iterative classifier. |
| `void` | `next(int iteration)`             Performs one iteration. |

**Method Detail**

### initClassifier

```
void initClassifier(Instances instances)
                    throws java.lang.Exception
```

:   Inits an iterative classifier.

    :   **Parameters:**: `instances` - the instances to be used in induction **Throws:**: `java.lang.Exception` - if the model cannot be initialized

---


### next

```
void next(int iteration)
          throws java.lang.Exception
```

:   Performs one iteration.

    :   **Parameters:**: `iteration` - the index of the current iteration (0-based) **Throws:**: `java.lang.Exception` - if this iteration fails

---


### done

```
void done()
          throws java.lang.Exception
```

:   Signal end of iterating, useful for any house-keeping/cleanup

    :   **Throws:**: `java.lang.Exception` - if cleanup fails

---


### clone

```
java.lang.Object clone()
                       throws java.lang.CloneNotSupportedException
```

:   Performs a deep copy of the classifier, and a reference copy
    of the training instances (or a deep copy if required).

    :   **Returns:**: a clone of the classifier **Throws:**: `java.lang.CloneNotSupportedException`


---


|  |  |  |
| --- | --- | --- |
| **Overview** \| **Package** \| **Class** \| **Tree** \| **Deprecated** \| **Index** \| **Help** \| **Weka's home** | |  |
| **PREV CLASS**   **NEXT CLASS** | **FRAMES**    **NO FRAMES**     **All Classes** |  |
| SUMMARY: NESTED \| FIELD \| CONSTR \| METHOD | DETAIL: FIELD \| CONSTR \| METHOD |  |


---
